# Effect of Plasma-Treated Water with Magnesium and Zinc on Growth of Chinese Cabbage

**DOI:** 10.3390/ijms24098426

**Published:** 2023-05-08

**Authors:** Rida Javed, Sohail Mumtaz, Eun Ha Choi, Ihn Han

**Affiliations:** 1 Plasma Bioscience Research Center, Applied Plasma Medicine Center, Kwangwoon University, Seoul 01897, Republic of Koreaehchoi@kw.ac.kr (E.H.C.); 2 Department of Plasma Bio-Display, Kwangwoon University, Seoul 01897, Republic of Korea

**Keywords:** plasma-treated water, nonthermal biocompatible plasma, Pak Choi, plasma agriculture, magnesium and zinc metal ions

## Abstract

Nonthermal biocompatible plasma (NBP) is an emerging technology in the field of agriculture to boost plant growth. Plasma is a source of various gaseous reactive oxygen and nitrogen species (RONS) and has a promising role in agricultural applications, as the long-lived RONS (H_2_O_2_, NO_2_^−^, NO_3_^−^) in liquid activate signaling molecules in plant metabolism. Plasma-treated water (PTW) has an acidic pH of around 3 to 4, which may be detrimental to pH-sensitive plants. Innovative techniques for producing PTW with a pH value of 6 to 7 under neutral circumstances are desperately required to broaden the application range of NBP in agriculture. Furthermore, Pak Choi (*Brassica campestris* L.) is a Brassicaceae family green vegetable that has yet to be investigated for its response to NBP. In this work, we proposed an alternate method for neutralizing the pH of PTW by immersing metal ions (Mg^2+^ and Zn^2+^) in the PTW and observing its effect on Pak Choi. After synthesizing PTW with MECDBD, we analyzed germination rate and growth parameters, then seedlings for 42 days to show physiological, biochemical, and molecular levels. The germination rate was observed to be higher with PTW and more efficient when metal ions were present. Seedling length and germination rates were dramatically boosted when compared to DI water irrigation. Because of the increased chlorophyll and protein content, the plants responded to the availability of nitrogen by generating highly green leaves. Furthermore, we observed that PTW increases the expression of NR genes and GLR1 genes, which are further increased when metals are submerged in the PTW. Furthermore, PTW and PTW with metals reduced ABI5 and CHO1 which is associated with a growth inhibitor. According to this study, nonthermal plasma might be utilized to significantly improve seed germination and seedlings’ development.

## 1. Introduction

Nonthermal biocompatible plasma (NBP) has been utilized in industry for many years, therefore its effectiveness in biomaterials is well known [1]. The NBP we used to be optimized to modulate target functions without causing damage to living cells or tissues. The application of NBP as a technique for treating plant material has also become more widespread in research. Before the turn of the century, research on NBP for seed and plant treatment served as the starting point and basis for the new field known as “Plasma Agriculture” along with expertise and knowledge obtained in plasma medicine [1,2]. The continual rise in food consumption caused by population growth demands such integrative study. In developing countries, the agricultural yield gap between real yields (obtained through field planting) and potential yields (obtained under controlled conditions for optimal plant development) can be as high as 50–75%. To ensure food security and economic growth in agricultural areas across the world, this large gap demands agricultural research. Traditional technological approaches such as irrigation, fertilizer, and crop protection were formerly used to increase productivity. The first stage in getting high output yields is to plant rapidly germinating, high-yielding seeds that produce healthy plants. As a result, chemical treatments are extensively used on seeds to protect them against diseases and pests and accelerate germination and fertilization. However, the impact of enhancing agricultural output through specific technology solutions typically overlooks the greater impact on the environment. 

NBP has recently emerged as an eco-friendly, cutting-edge technology in the universal seed culture method [1]. The plasma agricultural literature covers the efficacy of various NBP approaches, from seed to field [3,4]. Water treated with a plasma discharge has a significant influence on seed germination and seedling development in general. Plants that are irrigated with plasma-treated water (PTW) produce more agricultural output. PTW enhances the production of plant hormones such as auxin and cytokinin, as well as other physicochemical changes that improve plant germination, growth, and development, according to the literature [5,6,7]. Separately, extensive research has already been undertaken on the development and enhancement of plasma-assisted nitrogen fixation [7]. The RONS produced in PTW is a very important source of nutrients for a plant where they may function as signaling molecules in plant metabolism. Hydrogen peroxide can either diffuse freely across cell membranes or plants need membrane proteins called aquaporins to help water transport [8]. The nitrite and nitrate present in PTW are important sources of nitrogen for plants, and their transport across the plasma membrane occurs by diffusion or by specific transporter [9,10]. The composition of PTW has the potential to become an environmentally friendly and sustainable alternative to classic fertilizers in agriculture. To determine the impacts of specific RONS in PTW, it is required to better comprehend the process in plants. Previously, the impact of RONS was mostly determined by the plant growth indices and their overall appearance. Recently, the focus of research mainly investigated the microscopic and molecular level to better explore the physiological mechanism that RONS actuates in seeds and plants [11]. 

Moreover, the recent use of PTW has been expanded among plant physiologists because of its beneficial attributes [12,13,14]. PTW has been demonstrated to advance seed and seedling germination and plant advancement, limit the expansion of plant-related pathogenic microscopic bacteria, and treat fungus-infected seedlings [15,16,17,18,19]. PTW may significantly improve the germination and advancements of mung bean seeds [20]. PTW might enhance the drought tolerance and seed germination rate in radish and other crop seeds [21,22]. The nitrate and nitrite particles in PTW might play an essential role in the improvement of plant growth. The role of antibacterial enhances seed germination and plant growth by soaking seeds in PTW. The PTW might be utilized to promote crop yields against drought environmental circumstances [23,24]. 

Magnesium (Mg^2+^) and Zinc (Zn^2+^) are essential macronutrients for the improvement and development of plants and also play important roles in physiological and biochemical processes as well as providing plant defense mechanisms in abiotic stress [25,26,27,28]. The plant absorbs nitrate, ammonia, or both aggregate and the proportion of the two nutrients can maintain the cellular pH. In plants, the phloem is vital to tissue for food supply, mRNA, and signal transport, like a neural network associating the shoot and root [29,30]. Pak Choi (*Brassica campestris* L.) is a leafy vegetable of the Brassicaceae family; the main cultivar comes from China, and these days, it is cultivated worldwide. This plant is very important for human health due to the presence of beneficial compounds such as folate, vitamin C, carotenoids, phenolic compounds, and glycosylates [31,32,33]. The effect of PTW on Pak Choi is largely unknown. Despite many benefits, PTW has an acidic pH (~3–4) due to the dissolved nitrogen oxides and hydrogen peroxide. Soaking plants directly in such highly acidic PTW might be detrimental to crop output and also restrict the usage of NBP on plants that are sensitive to pH. As a result, additional approaches for generating PTW with a pH value of (6–7) under neutral conditions are necessary to expand the application range of NBP in agriculture [34]. 

In this work, we developed an alternate method for neutralizing the pH of PTW by immersing metal ions (Mg^2+^ and Zn^2+^) in the PTW. Furthermore, we investigated the effect of PTW with neutralized pH on Pak Choi for the first time. The air, metal, and water are used to produce a liquid fertilizer utilizing a multi-electrode cylindrical dielectric barrier discharge (MECDBD), which shows great potential for linking with on-site growth systems for the on-demand application. The method can be linked to soil-based or hydroponic growing systems. To adjust the acidity of the PTW, several important metals (M: Mg^2+^, Zn^2+^) were dissolved. It was discovered that these metals not only reduced the acidity of the PTW but also increased the rate of nitrogen fixation. We first examined seed germination rate and growth characteristics after preparing PTW with MECDBD, then seedlings for 42 days to reveal physiological, biochemical, and molecular levels.

## 2. Results

### 2.1. Electrical and Optical Properties of MECDBD Plasma Device

**Figure 1:**
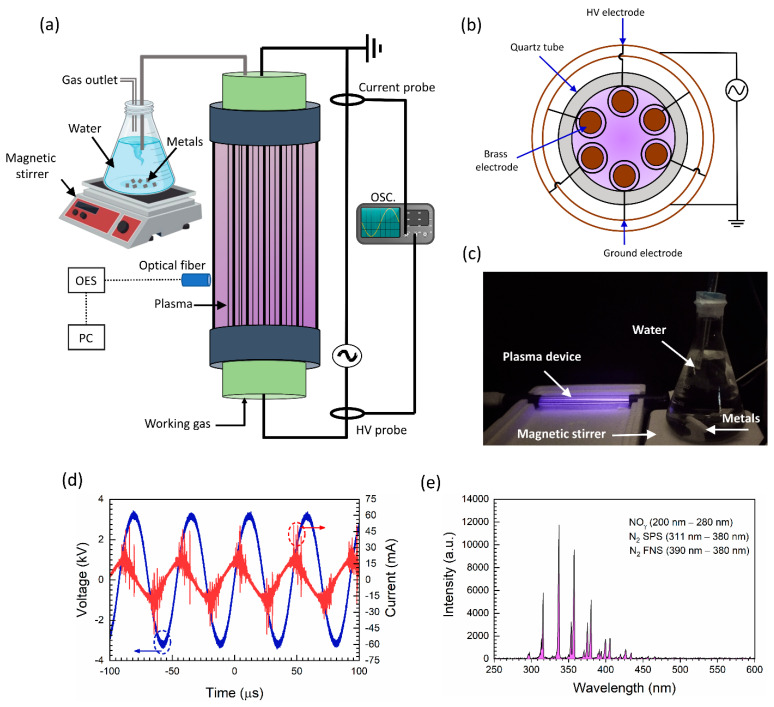
Experimental setup of multi-cylindrical DBD plasma source: (**a**) Schematic; (**b**) cross-sectional view; (**c**) photograph of the plasma source; (**d**) voltage and current waveform; (**e**) optical emission spectra of MECDBD.

Figure 1 shows the experimental setup and properties of MECDBD. Moreover, we also examined the Pak Choi seedlings at the physiological, biochemical, and molecular levels. The device MECDBD, which is used to generate plasma and experimental arrangements, is shown in Figure 1a. The cross-sectional view of the device is displayed in Figure 1b, where three electrodes were connected to high voltage, and the remaining three were connected to the ground. Figure 1c shows the photograph of the MECDBD while preparing PTW.

Figure 1d shows the current-voltage waveform of MECDBD. The discharge current peaks that occur in each half cycle of the applied voltage cause the voltage waveform to seem distorted. With a rise in applied voltage during the applied voltages positive half cycle, a positive current peak develops. During the negative half cycle, certain charges that have been collected within the dielectric substance are reversed. As a result, the applied voltage’s negative half cycle experiences a peak in the negative discharge current. Here, the discharge current is 25.0 mA, the applied voltage is 3 kV, and the frequency is 20 kHz as described in Figure 1d. The optical emission spectrum (OES) of the MECDBD is shown in Figure 1e when the air is feeding gas. The intensity of the NOγ bands exists between 200 and 280 nm. While using the ambient air as a working gas, the energetic electrons collide with nitrogen and oxygen to produce the reactive species in contact with the water’s surface. However, according to the OES peaks of the plasma source, the nitrogen second positive system (N_2_ SPS) is seen between 311–380 nm. The emission from the nitrogen first negative system (N_2_ FNS) was observed between the wavelength range of 390–440 nm [35].

### 2.2. Physiochemical Characteristics of Irrigation Seedlings

The MECDBD plasma source is intended to generate mostly RNS in water. The quality parameters (pH, ORP, EC, and TDS) of water were measured in all conditions used to irrigate the Pak Choi seedlings. For treatment conditions: control (DI), DI + M (no str.), DI + M, (str.), PTW (str.), and PTW + M (str.), the pH was measured and displayed in Table 1. It was observed that the pH was significantly decreased up to 3.9 in PTW while combining the metals with plasma gave a neutralized pH (similar to DI) of PTW. The metal ions, nitrites, and nitrates were produced during the experiment as an essential role in seedlings’ growth and plant development. PTW (str.) is also well known to enhance the seed germination rate and plant growth due to the source of RNs. Moreover, during plasma treatment with the addition of metals, there is more significant irrigation due to the controlled pH and also essential metals nitrate and nitrite with the presence of RONS formed.

The major chemical properties such as oxidation-reduction potential (ORP) and electric conductivity (EC) are listed in Table 1. Furthermore, total dissolved solids are the most significant for measuring the physiochemical aspects of irrigation because they characterize the ion concentration and redox environment of the water solution when compared to control treatment conditions. In addition, the amounts of H_2_O_2_ and NO_x_ in water treated under various conditions were tested, and the results are presented in Table 1. The H_2_O_2_ levels were observed to be slightly higher in plasma treatment while remaining non-significant in all other treatment conditions, while the NOx content was found to be considerably enhanced in PTW (str.) as well as in PTW + M) (str.).

### 2.3. Seed Germination and Growth Parameters of Pak Choi

**Figure 2:**
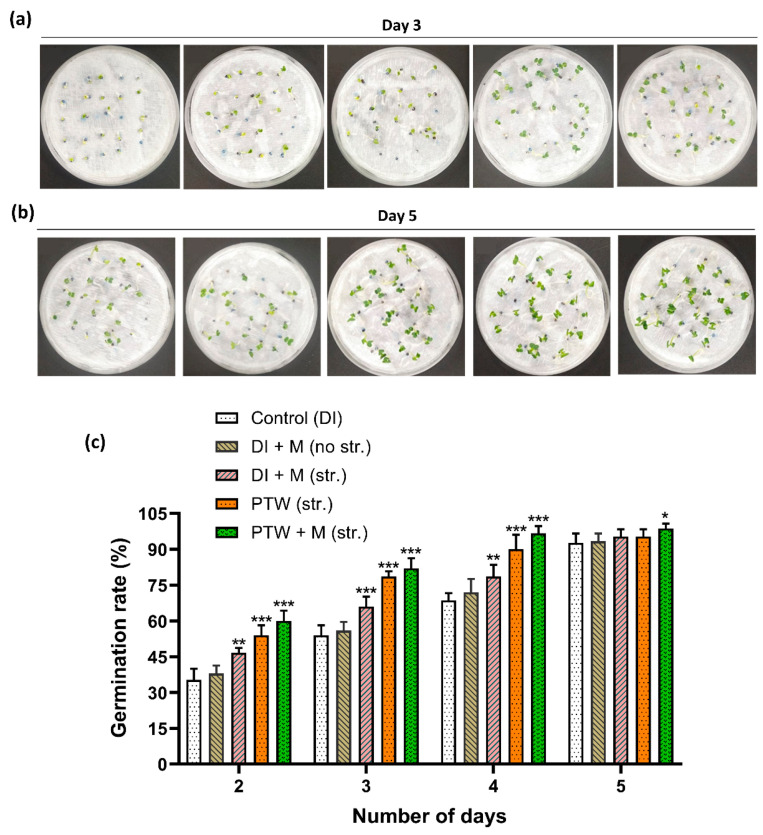
Germination of Pak Choi seeds after sowing on a paper towel. Photograph of seedling growth on the (**a**) 3rd and (**b**) 5th day after sowing, (**c**) average germination rate. Data are the mean ± SEM of the triplicate experiment in duplet, statistical analysis was done by the student’s *t*-test *p*-value denoted by * *p* < 0.05, ** *p* < 0.01, and *** *p* < 0.001.

The germinate rate of Pak Choi seeds was examined from each day subsequently with all irrigation conditions. The germinated seeds of Pak Choi were considered when the radical was around 2 mm in length. The photographs of day 3 and day 5 described the results of seedlings after sowing on wet cotton with control (DI), DI + M (no str.), DI + M (str.), PTW (str.), and PTW + M (str.) throughout the 5 days as shown in Figure 2a,b. Early seed germination days have a very important role in observing the germination rate. In the 24 h after sowing (day 2), the germination rate was 35%, 38%, 46%, 54%, and 60% in the control (DI), DI + M (no str.), DI + M (str.), PTW (str.), and PTW + M (str.), respectively. On day 3, the germination rate was 54%, 56%, and 66% without plasma, and it was 78% and 82%, respectively, in the PTW (str.) and PTW+ M (str.). Following the treatment sequence shown in Figure 2, the germination rate on day 4 was 68%, 72%, 78%, 90%, and 96%, and on day 5 it was 92 %, 93%, 95%, 95%, and 98%. The germination rate for each day is plotted in Figure 2c. According to the results of seed germination, positive control DI + M (str.) has significant but PTW and PTW + M (str.) had a more significant rate of substant days. The more detailed seed germination comparison with treatments concerning days is illustrated in such as Supplementary Materials Table S1.

**Figure 3:**
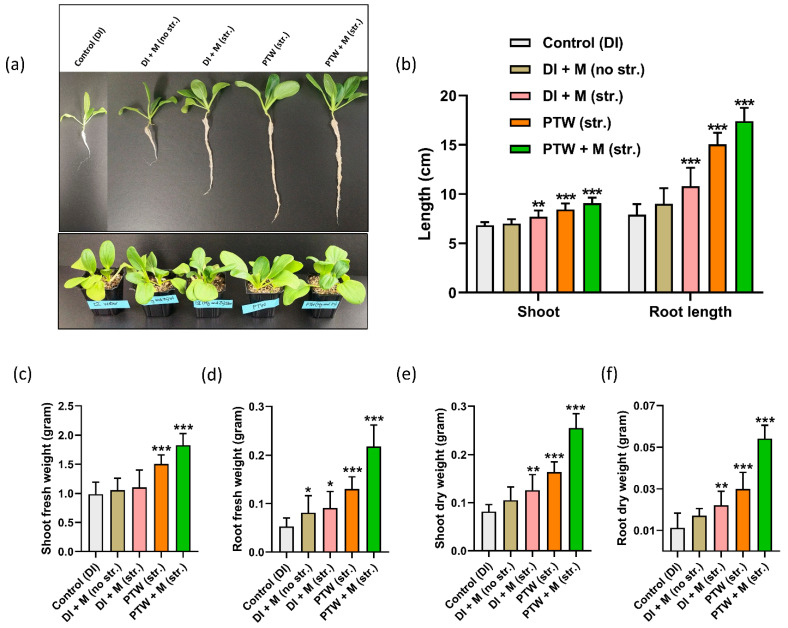
The physiological status of Pak Choi seedlings: (**a**) photograph of Pak Choi (42 days old) plants grown in vermiculite; (**b**) shoot and root length (cm); the average fresh weight of (**c**) shoot including leaves; (**d**) root; the average dry weight of (**e**) shoot; (**f**) root in gram in control (DI), DI +M (no str.), DI + M (str.), PTW (str.), and PTW + M (str.). The statistical analysis was done by the student’s *t*-test, *p*-value denoted by the * *p* < 0.05, ** *p* < 0.01, and *** *p* < 0.001.

To better understand the growth and development of Pak Choi plants, after 42 days of irrigation with various water, they were carefully separated from vermiculite. Figure 3a shows an image of seedling. The shoot and root length of every seedling watering was measured. On the 42 days, average shoot lengths including leaf were 6.84 cm with the control (DI), 7.25 cm with DI + M (no str.), 7.71 cm with DI + M (str.), 8.44 cm with PTW (str.), and 9.07 cm in PTW+ M (str.). The root length was also measured, and the result is indicated in Figure 3b. For treatment conditions: control (DI), DI + M (no str.), DI + M, (str.), PTW (str.), and PTW + M (str.), the root length was measured as 7.9, 9.2, 10.81, 15.06, and 17.41 cm, respectively. The addition of metals within water improved root length slightly, but large increases were identified with the presence of plasma, and further increases were observed when pH was neutralized with metals in PTW + M (str.). 

The fresh weight of the shoot and root of Pak Choi varied due to the quantity of water absorbed. For the following treatment conditions: control (DI), DI + M (no str.), DI + M (str.), PTW (str.), and PTW + M (str.), the fresh weight of the shoot was measured as 0.998, 1.0567, 1.10, 1.50, and 1.82 g, and for the root, it was determined as 0.05 g, 0.08 g, 0.09 g, 0.13 g, and 0.217 g, respectively, and findings are given in Figure 3c,d. Due to the presence of water, measuring the dry weight of the shoot and root is more accurate than the fresh weight. Furthermore, the dry weight of the shoot was determined to be 0.081, 0.10, 0.126, 0.163, and 0.255 g, and the dry weight of the root was obtained as 0.0111, 0.017, 0.022, and 0.03 g, under the following treatment conditions: control (DI), DI + M (no str.), DI + M (str.), PTW (str.), and PTW + M (str.) as given in Figure 3e,f. It was discovered that the development in fresh and dry weight of Pak Choi shoot and root follows a comparable enhancement pattern with the corresponding treatment conditions.

The diameter of the shoot and root was also measured, and results are provided Such as Supplementary Materials in Figures S1 and S2. The average leaf number was also counted in all irrigated conditions, and the maximum leaf number was counted in the PTW (str.) and PTW + M (str.), and results are provided in Supplementary Materials in Figure S3. Based on the results of all experiments, it can be noticeably concluded that neutralizing PTW with metal ions not only enhances the seed germination rate but also increases plant growth and development.

### 2.4. Biochemical Profiling in the Pak Choi Seedlings

**Figure 4:**
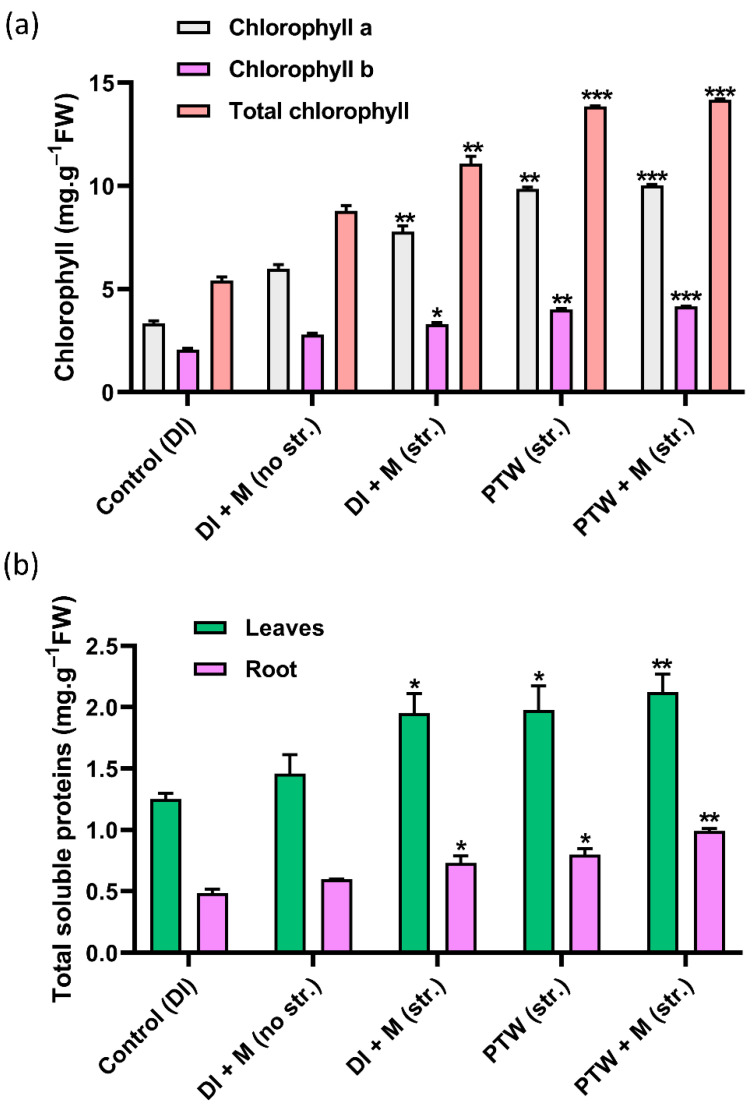
(**a**) Chlorophyll detection in fresh leaves and (**b**) total soluble protein in fresh leaves and roots of Pak Choi seedlings grown in control (DI), DI + M (no str.), DI + M (str.), PTW (str.), and PTW + M (no str.). The results shown in terms of error bars and significant difference were examined by the student’s *t*-test, *p*-value symbolized by * *p* < 0.05, ** *p* < 0.01, and *** *p* < 0.001.

The photosynthetic efficiency correlates with the chlorophyll levels in leaves. The chlorophyll content was measured in fresh leaves after 42 days of soaking in different irrigation such as Supplementary Materials in Figure S4. The trend of increasing deep green color was observed and compared and the results are displayed in Figure 4a. In the following treatment conditions: Control (DI), DI + M (no str.), DI + M (str.), PTW (str.), and PTW + M (str.) chlorophyll a content is measured as 3.34, 5.99, 7.78, 9.85, and 10.01 mg/g; chlorophyll b is 2.06, 2.78, 3.29, 4.01, and 4.16 mg/g; and total chlorophyll is 5.41, 8.80, 11.07, 13.85, and 14.17 mg/g, respectively. The plants fixed the nitrogen content in the form of nitrite and nitrate and some content of reactive nitrogen and hydrogen species irrigated with PTW (str.) and PTW + M (str.) has essential compound magnesium nitrate and magnesium nitrite and zinc nitrate and zinc nitrite along with the reactive nitrogen and hydrogen species. These are all mainly responsible for the increased plant growth and development. 

The total soluble protein was measured in the fresh leaves and roots of Pak Choi seedlings irrigated with all treatment conditions. Total soluble protein was determined to be 1.25, 1.46, 1.95, 1.97, and 2.12 mg/g in fresh leaves and 0.48, 0.59, 072, 0.80, and 0.99 mg/g in roots under the following treatment conditions: control (DI), DI + M (no str.), DI + M (str.), PTW (str.), and PTW + M (str.), respectively, and the results are shown in Figure 4b. Soluble protein levels were found to be substantially higher in fresh leaves and roots in PTW alone, and even higher when metals were added to the PTW. Therefore, further experiments were conducted by using the PTW and PTW + M treatment conditions.

### 2.5. Endogenous RONS in Pak Choi Seedlings

**Figure 5:**
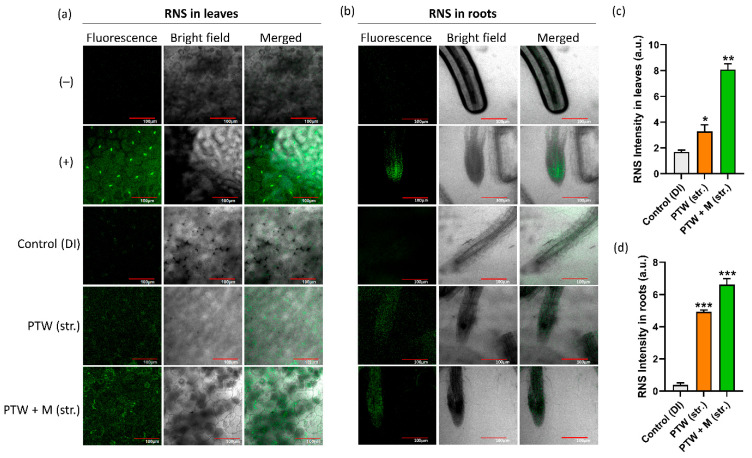
Microscopic endogenous NOx detection in Pak Choi seedlings (**a**) leaves and (**b**) root tips by using a confocal microscope. The fluorescence intensity of RNS in (**c**) leaves and (**d**) roots was plotted by Image J software. The statistical analysis was done by the student’s *t*-test, with *p*-value denoted by the * *p* < 0.05, ** *p* < 0.01, and *** *p* < 0.001.

The endogenous RONS concentration, H_2_O_2_, and NO_x_ were directly determined in the leaves and roots of PTW and PTW with metals, and the results were compared with the control (DI). The RNS content was measured in vivo for leaves and roots of Pak Choi seedlings using a fluorescent dye 4-Amino-5-Methylamino-2′,7′-Difluorofluorescein Diacetate (DAF-FM Diacetate) and fluorescent images were captured by the confocal microscopy. The RNS concentration of PTW leaves grew considerably, but more strongly in PTW with metals than in control DI leaves, as seen in Figure 5a. Similarly, the RNS content was also measured in roots. Figure 5b shows that the RNS concentration in the root by PTW has increased significantly, but more strongly in PTW with metals than in control. The fluorescent intensity of RNS detection in leaves and roots in plasma treatments was plotted as compared with control (DI), as shown in Figure 5c,d, respectively. These results show that PTW (str.)- and PTW + M (str.)-induced reactive nitrogen species positively influence the endogenously RNS status of seedlings.

**Figure 6:**
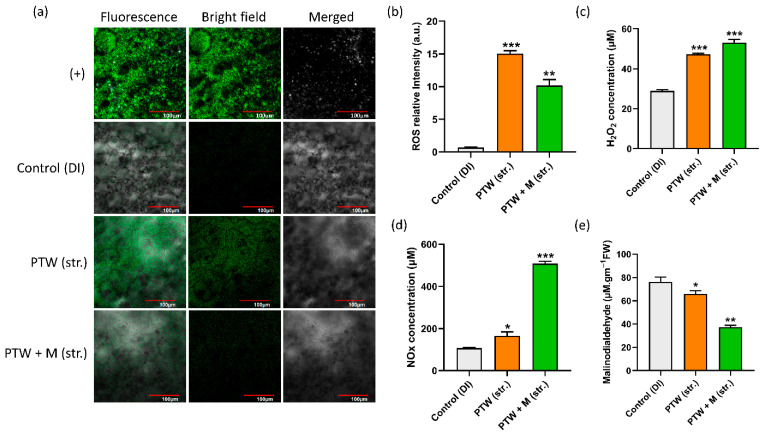
Estimation of (**a**) Microscopic ROS detection in Pak Choi leaves, (**b**) ROS fluorescence intensity. Spectrophotometric quantitation of (**c**) H_2_O_2_ and (**d**) NOx. Lipid peroxidation detection by the (**e**) Malondialdehyde in Pak Choi leaves. The statistical analysis was done by the student’s *t*-test, *p*-value denoted by the * *p* < 0.05, ** *p* < 0.01, and *** *p* < 0.001.

The ROS level was also detected in the leaves by the H2DCFDA fluorescence dye. The obtained findings reveal that the treatment conditions PTW (str.) have a greater quantity of ROS than the control; however, in the case of PTW + M (str.), ROS remains higher than the control but decreases somewhat when compared to PTW alone, as shown in Figure 6a. The ROS fluorescence intensity was shown in Figure 6b. The ROS intensity level was increased in PTW (str.) leaves as compared to PTW + M (str.). Moreover, to check the quantification of RONS levels in the leaves separately, the quantification results of H_2_O_2_ concentration in the control were determined to be 28.9 µM, whereas in PTW alone and PTW with metals were determined to be 47.2 µM and 53.09 µM, respectively, as shown in Figure 6c. The quantification results of NO_X_ concentration in the control were determined to be 107.02 µM, whereas in PTW alone and PTW with metals were determined to be 165.3 µM and 508.37 µM, respectively, as shown in Figure 6d. A well-known stress marker for membrane damage caused by PTW (str.)-induced reactive species as well as neutralized PTW + M (str.) is malondialdehyde (MDA). The MDA content was found to be marginally reduced with PTW and significantly reduced in PTW with metals. The MDA concentration in the control was determined to be 76.09 µM, whereas in PTW alone and PTW with metals was determined to be 65.92 µM and 37.27 µM, respectively, as shown in Figure 6e. In our results, the MDA content was decreased which showed that there is no membrane damage occurred by the PTW and PTW + M (str.). Moreover, the high level of MDA hindered the PSII core protein and Rubisco which are important enzymes of the Calvein cycle during respiration [36].

### 2.6. Plasma-Induced Upregulated Plant Growth Genes at the Molecular Level

**Figure 7:**
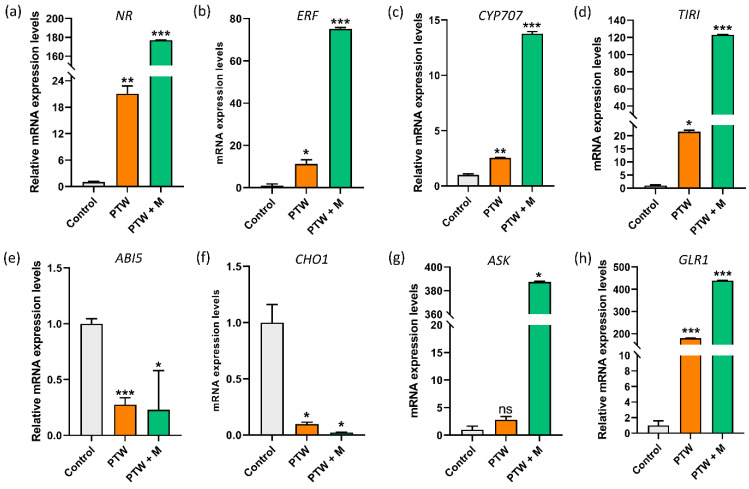
The relative gene expression by q-rt PCR of the different growth-related genes of nitrate signaling (**a**) NR, (**b**) ERF, (**c**) CYP707, (**d**) TIRI, and growth inhibitor by ABA degradation as (**e**) ABI5, (**f**) CHO1, resultantly, activation of growth-promoting genes (**g**) ASK and (**h**) GLR1, respectively in Pak Choi leaves. The statistical analysis was done by the student’s *t*-test, *p*-value denoted by the * *p* < 0.05, ** *p* < 0.01, and *** *p* < 0.001.

The expression of several growth genes associated with NO signaling with different nitrogen forms nitrate and nitrite to maintain the nutrient balance was analyzed by q-RT PCR to understand the effect of PTW (str.) and PTW + M (str.) on dormancy break led to the enhancement of seedling growth and development. NO as external stimuli inhibited the vacuolation and increased the aleurone cell synthesis around the testa [37]. The dormancy break and seedling growth were maintained by the NR which was up-taken in the form of nitrate, which modulates group V II ERFs as transcriptional regulators via the targeted proteolysis pathway in plants [38]. The NR genes which have a major role in plant growth and development have significantly increased in the PTW and more increased in the PTW with metals as compared to control leaves (Figure 7a). 

Moreover, expression of the ERF gene was activated in PTW alone and increased in PTW with metals (Figure 7b). The CYP707A2 seedling growth gene is involved in ABA catabolism by maintaining endogenous nitrate, which is associated with the s-nitrosylation of TIRI that has a positive effect on the auxin signaling [39]. The CYP707A2 expression more significantly increased in the PTW (str.) and PTW + M (str.) leaves and increased significantly compared with the control (DI) (Figure 7c). The TIRI growth genes or transport inhibitor response 1 protein involved in the auxin biosynthesis expression of TIRI also significantly increased in the PTW (str.) leaves and more significantly activated in the PTW + M (str.) leaves (Figure 7d). The ABI5 and CHO1 genes are inhibitors that constrain plant growth and increase dormancy [40]. In this work, the expressions of ABI5 and CHO1 genes were found to be decreased in both cases: PTW alone and PTW with metals (Figure 7e,f). The mRNA level of ASK was induced in leaves by PTW and PTW + M as shown in Figure 7g. The mRNA of putative glutamate receptor1 (GLR1) increased in PTW (str.) as well as further increased expression level observed in the PTW + M (str.) in Figure 7h. In the present study, PTW and PTW induced the expression of growth genes particularly, NR and ERF expression showing the regulatory effect on auxin biosynthesis and ABA catabolism.

All these results indicate that PTW and PTW + M irrigation of Pak Choi seedlings induced several physiological, biochemical, and molecular level changes related to hormones. The activation of the gene by PTW alone improved plant growth and the inclusion of metals in PTW increased it even further.

## 3. Discussions

This study aimed to examine the impact of PTW (str.) and PTW+ M (str.) irrigation on the seedling growth gene mechanism. The enhanced seedling growth mechanism related to RONS is produced by plasma treatment. The impact of PTW (str.) and PTW+ M (str.) irrigation on the seedlings growth of Pak Choi has been observed in relation to the generation of RONS by plasma treatment. The enhanced seedling growth mechanism related to RONS is produced by plasma treatment. The air as a feeding gas generated various RONS as compared to other gases such as argon, N_2_, and O_2_ [41]. The main RNS species were detected in the OES of the MECDBD. These species influence the physiochemical characteristics of water by interacting with its molecules [42]. These RONS influence plant growth by altering the chemical composition of PTW and lowering its pH. In the agricultural prospectus, using metals in PTW has advantages [7]. Metals modify the PTW into neutral by removing H^+^ ions and turning them into nascent hydrogen. The presence of metal ions in the water causes beneficial physiological changes in plants [43]. In this work, magnesium and zinc metals were used during plasma treatment to neutralize the pH. 

Moreover, nitrogen and Mg are essential for growth and production, so sustained food quality is thus required at sufficient levels, particularly for plant growth and development [44]. Apart from that, Zn is a crucial essential micronutrient that influences a few metabolic processes and co-factor of various enzymes [45]. The effect of PTW with low pH enhanced the seedling growth of the radish (*Raphanus sativus*), tomato (*Solanum lycopersicum*), and sweet pepper seedlings (*Capsicum annum*) due to the NOx concentration [21]. Furthermore, nitrate and nitrite ions concentration promote seedling growth as nitrogen fertilizer [24]. Mostly, higher availability of nitrogen results in improved plant productivity. Numerous nitrogen-sensitive membranes make up plants and fixed these nitrogenous compounds from the soil. Plants that respond to nitrogen undergo morphological and physiological changes that lead to enhanced agricultural yields. The rhizosphere absorbs fixed nitrogen and metal ions, which are then distributed to root transporters and then assimilated by the various plant parts [36]. The nitrogen content, metal ions, and pH value limits can change depending on the target liquids’ volume and treatment period. However, longer plasma treatment has had an adverse effect on plant growth and development due to a higher generation of RONS [46]. Plants can become stunted by excessive metal ions which adversely affect the metabolic and enzymatic function. On the other hand, present studies investigated that optimized dissolved metal ions in PTW had increased germination and seedling growth. Metal ions can improve cellular metabolism and act as a catalyst to accelerate the production of protein and chlorophyll [47]. In previous studies, researchers found that PTW pH must be neutral due to irrigation with acidic water can harm the plants by causing root burn. 

In the present study, qualitative and quantitative H_2_O_2_ and NOx levels were increased in the Pak Choi seedlings by PTW alone and PTW with metals irrigation. These results are similar to the endogenous H_2_O_2_ content of *Capsicum annum* plants stimulated by the external application of 14 mM and 18 mM H_2_O_2_ solutions. The H_2_O_2_ and NO as RONS cause signaling molecules in plants [48]. Therefore, it is suggested that H_2_O_2_ and NO_x_ solutions have similar effects on plant cells and comparable mechanisms of action [49,50].

In this work, transcriptional regulation of H_2_O_2_ and NOX generated by plasma with and without metals in Pak Choi seedling’s growth showed that RONS mutually regulate the expression of nitrate signaling networks and their involvement in seedling growth. NR is a nitrate reductase gene that breaks dormancy [51]. The regulation of nitrate absorption at the post-translational level was also examined. Furthermore, nitrate reductase mutant G’4-3 of Arabidopsis was even less inactive than the wild type with barely 0.5% of the nitrate reductase activity of the equivalent wild type [39]. NO derived from NR activity affects ERF transcriptional regulators’ stability by targeted proteolysis and changes in NR levels during plant growth modify NO levels and would link metabolism to gene regulation [52]. For exogenous nitrate to effectively overcome the seed dormancy, regulation of ABA degradation is more important than the ABA synthesis. They proposed that endogenous nitrate may regulate the seed ABA content through the activity of the ABA catabolism gene CYP707A2. Additionally, as CYP707A2 mutant seeds behaved like the wild type under 10 mM nitrate, other genes such as NCED9 must be involved in the modulation of dormancy by nitrate [53]. Furthermore, NO stimulates auxin signaling via S-nitrosylating TIRI [40]. The gene CHO1 encodes a putative transcription factor that has two AP2 domains and is mostly expressed in seeds, impacted by an excess of glucose and nitrate levels [54]. The present and previous studies activate enhanced seedling growth by nitrate and ABI5 signaling by NO_X_ and H_2_O_2_ activation as external stimuli. Overall results investigate that PTW (str.) and PTW + M (str.) can stimulate both plant growth and dormancy break with nitrate and ABI5 signaling mechanism. A better understanding of the plasma effect generated by RONS signaling in plants must be prioritized in the future. 

## 4. Material and Method

### 4.1. Plasma Device (MECDBD) and Experimental Setup

**Figure 8:**
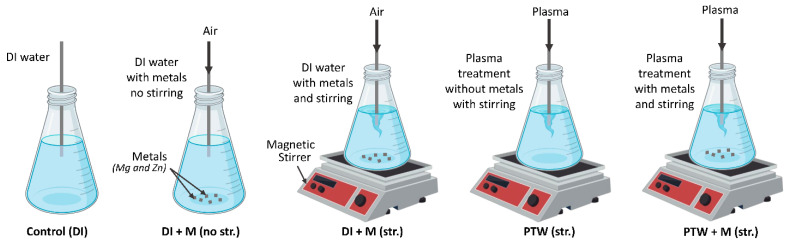
The schematic of different treatment conditions used to prepare water for Pak Choi seedlings.

In this work, the MECDBD was used to generate plasma as shown in Figure 1a. The device was designed by using multi-six) brass electrodes and each electrode was inserted inside an (inner) quartz with an inner diameter of 20 mm. The diameter of each brass electrode was 20 mm, which is similar to the inner diameter of (inner) quartz. The brass electrode and (inner) quartz were 170 mm in length. Finally, all electrodes and (inner) quartz were inserted inside the outer quartz having an inner diameter of 90 mm and 25 cm in length. From six brass electrodes, three were connected to a high-voltage AC source and three were connected to the ground as shown in Figure 1a. The air was used as a feeding gas. When an AC source (3 kV, 20 kHz) was applied to air, plasma was generated inside the tube dissolved in water. The generated plasma was connected with the plastic pipe which was immersed in 1000 mL of deionized (DI) water to prepare PTW for 1 h treatment time with 450 rpm continuous stirring. Rather, it is the afterglow gas, enriched with chemical species, which is touching the water and exchanging with it. For pH neutralization, the metal ions (Mg^2+^ and Zn^2+^) which are represented as “M” with a weight of 4 g were used when preparing water. The size of magnesium metal was 1 cm and 2 mm in thickness. The zinc was 2 cm long and 0.1 mm in thickness. For further experiments, the water was prepared in five different conditions which were as follows: control (DI only), negative control DI + M (no str.), positive control DI + M (str.), PTW (str.), and PTW + M (str.). Moreover, the plasma treatment time in both conditions was 1 h, with the same stirring at 450 rpm. Here, str. Denotes the use of magnetic stirring when making PTW, and M denotes the addition of metal ions (Mg^2+^ and Zn^2+^) at a 50% ratio each. In this work, we determined the different seedling rates while treating with water prepared in five different conditions as shown in Figure 8.

### 4.2. Preparation of Irrigation and Its Physiochemical Characteristics

In this work, there were five total water conditions of which three were controls, such as control (DI), and the other two were positive and negative controls of metals such as DI + M (str.), and DI + M (no str.), in the presence of air gas, respectively. The remaining two were plasma treatments such as PTW (str.) with an exposure time of 60 min and an airflow rate of 6 L per minute (lpm) and PTW + M (str), together with the same parameters. The pH, oxidation reduction potential, conductivity, and total dissolved solids were measured by the portable OAKTON PCTS TESTR™ 50 as a function of physiochemical properties. The RONS detection was carried out with the QuantiChrom^TM^ NO assay kit and QuantiChrom^TM^ peroxidase assay kit (Bioassay Systems, Hayward, CA, USA) which measured the amount of total NO_x_ and H_2_O_2_ in the sample from 540 nm and 585 nm absorbance by using the Bio Tek Gen 5 microplate reader, respectively [55,56].

### 4.3. Germination and Seedling Growth Parameters

Pak Choi (*Brassica rapa subsp. Chinensis* L.) was used as a model seed to find out the impact of PTW (str.), and PTW + M (str.) on the status of genes level. In Petri dishes with cotton covering, trials of 105 seeds were put (each Petri dish contained 25 seeds) in a triplicate doublet experiment. Each seed trial was irrigated with 2 mL control (DI), DI + M (no str.), DI +M (str.), and PTW (str.), PTW + M (str.) respectively. The seed trials were maintained in a plant growth chamber at 25 ± 2 °C and 76 ± 5% relative humidity, and a 16–8 h light–dark cycle (irradiance 44 W m^−2^, Philips Fluorescence Tube Light Bulb). The seed germination status was every day until five, and the germination rate was calculated from the equation [21].

$$Germination\;rate=\frac{Number\;of\;germinated\;seed}{\mathrm{Total}\;\mathrm{number}\;\mathrm{of}\;\mathrm{seeds}}\times 100\%$$

For the plant growth analysis, another 20 seeds were used for each set of trials. Firstly, equally germinated seeds in DI water were planted in 50 g of vermiculite in the triplicate experiment. Each selected pot was the same size and two equally germinated seeds for each set of trials were put in vermiculite. After this, the Pak Choi seedlings were irrigated with 10 mL of each condition freshly prepared PTW (str.), and PTW + M (str.), twice a week. At the end of six weeks (42 days old seedlings) then, the plants were gently pulled out from the vermiculite, and various physiological parameters including length, fresh weight, and dry weight, of shoot and root length, respectively, were measured. Moreover, the shoot and root diameters of Pak Choi plants were measured by using the micrometer screw gauge. The number of leaves was also counted in each condition.

### 4.4. Biochemical Assay

#### 4.4.1. Chlorophyll Content

For the determination of chlorophyll content, each sample of two to three fresh leaves was cut into small pieces and thoroughly mixed. Weighing and transferring a sample of 200 mg into a 25 mL test tube was followed by adding 80% acetone. To make sure all leaves were cleaned with acetone solution, the test tube was turned up and down several times. After covering, the test tube with aluminum foil, it was incubated at room temperature for 3–4 days up to transparent leaves. The extracted liquid was filtered, and the absorbance of 200 µL of the liquid deposited in a 96-well plate with a transparent bottom was measured at the wavelength of 663 and 645 nm, with the help of a plate reader (Synergy HTX Multi-Mode Reader from Bio Tek Instruments, Winooski, VT, USA). The amount of chlorophyll a, chlorophyll b, and total chlorophyll were calculated from the equation [57].

(1) $$Chlorophyll\;a=\left(12.27\times A663.2\right)-\left(2.79\times A646.8\right)\;$$

(2) $$Chlorophyll\;b=\left(21.50\times A646.8\right)-\left(5.10\times A663.2\right)\;$$

(3) Total Chlorophyll=chlorophyll a+chlorophyll b 

#### 4.4.2. Total Soluble Protein Detection

Fresh leaf and root samples weighing 0.2 g were immediately frozen in liquid nitrogen. Resultantly, for the protein analysis, the powdered dust of samples was mixed with 1 mL of 1× RIPA buffer and centrifuged at 20,000× *g* for 15 min. The ice bath was used to keep this solution cool until complete decantation. After that, the supernatant was moved to another test tube. Using the DC protein assay (Bio-Rad, Hercules, CA, USA) method and bovine serum albumin as a reference, the amount of total protein was measured [58].

#### 4.4.3. Malondialdehydes Detection

Malondialdehyde concentration in plants is a direct measure of lipid peroxidation induced by the RONS. MDA is well known stress marker to determine the membrane damage induced by plasma treated water. The Pak Choi seedlings samples weighing 0.5 g were powdered by using liquid nitrogen and were mixed with 4 mL of 20% Trichloroacetic acid containing 0.5% Thiobarbituric acid. The homogenized samples were incubated at 95 °C in a water bath for 15 min. Samples were immediately transferred to an ice bath for cooling and centrifuged at 10,000× *g* for 10 min. The MDA standards and samples should be transferred in 150 µL to a 96-well black fluorescence microplate that can be read by a fluorometric plate reader. MDA fluorescence was measured at 540 nm excitation and 590 nm emission, the sample was read and standard in triplicate [59].

### 4.5. Detection of Hydrogen Peroxide and Nitric Oxide Assay Seedlings

Reactive oxygen and nitrogen species were in situ visualized in the leaves of Pak Choi seedlings to examine the impact of PTW and PTW with metals on the redox levels of the plants. Using the QuantiChrom ^TM^ Peroxide Assay Kit, endogenous H_2_O_2_ of Pak Choi seedlings PTW and PTW + M (str.) was discovered spectrophotometrically (Bioassay Systems, USA). In this assay, the formation of a purple color complex (Fe^3+^-xylenol orange reaction) followed the oxidation of Fe^2+^ into Fe^3+^ by H_2_O_2_. The endogenous seedlings were irrigated with PTW and PTW + M (str.)) NO_x_ amounts were measured by the QuantiChrom^TM^ NOx Assay Kit [60]. The in situ reactive nitrogen species and reactive oxygen species were determined by the DAF-FM Diacetate (4-Amino-5-methylamino-2,7-Difluorofluorescein Diacetate) as well as H2DCFDA fluorescence dye, respectively. According to this protocol, the leaves were excised freshly and cleaned with distilled water. Peeling off the abaxial epidermis of leaves was done before they were submerged in 10 M of DAF-FM (made in 10 mM MES-KCl solution) for two hours and placed at room temperature. Following incubation, leaf samples were completely cleaned with 10 mM MES-KCl buffer before being examined using an Olympus IX83-FP confocal microscope (Olympus, Tokyo, Japan) with epi-fluorescence Alexa fluor 488 filters (excitation 495 nm and emission 515 nm). The leaves and roots of Pak Choi were irrigated with distilled water pretreated with 100 µM sodium nitroprusside and 100 µM cPTIO for 2 h during RNs detection. For the H_2_O_2_ in leaves, the H2DCFDA fluorescence dye was used; Pak Choi leaves for positive control were pretreated with 10 µM H_2_O_2_ for 2 h [61].

### 4.6. qRT-PCR for Measuring Gene Expression Profiling

Quantitative PCR was used to analyze the expression of several growth-related genes in Pak Choi seedlings. Using the web program Primer 3, the primers utilized in gene expression analysis were created as shown such as Supplementary Materials Table S2. According to the established methodology, 500 mg of frozen leaf samples were utilized for total RNA extraction using RNAiso Plus reagent from Takara Bio Inc., Kusatsu, Japan. cDNA was made from 2 µg of RNA using oligo dT primers. Using the enzynomics M-MLV Reverse Transcriptase qPCR RT kit, the total RNA concentration was determined using a spectrophotometer and transformed into cDNA. Utilizing SYBR green chemistry, real-time PCR was carried out using a CFX96TM Real-Time System (BioRad) thermocycler, SYBR green master mix, molecular grade water, twentieth-fold dilution, and primers (10 µM each). The thermocycler program was as follows, and 96-well Bio Rad plates were used for the PCR reaction: Initial denaturation at 95 °C for 10 min, denaturation at 10 s, annealing at 60 °C for 15 s, and extension at 72 °C for 20 s. The superscript $\;2^{-\Delta CtCt}$ values for the expression were computed after an analysis of the qPCR data. A duplicate experiment of each reaction was carried out.

## 5. Conclusions

Nonthermal biocompatible plasma (NBP) is a new agricultural technique that stimulates plant growth. The acidic pH of PTW (3–4) may be harmful to pH-sensitive plants. To widen the use range of NBP in agriculture, novel strategies for generating PTW with a pH value of (6–7) under neutral conditions are critically needed. We presented an alternative approach for neutralizing the pH of PTW in this study by immersing metal ions (Mg^+2^ and Zn^+2^) in the PTW and evaluating the impact on Pak Choi. We studied seed germination rate and growth characteristics after synthesizing PTW using MECDBD, then seedlings for 42 days to exhibit physiological, biochemical, and molecular levels. When metal ions were present, the germination rate and plant growth were shown to be greater with PTW and more efficient. Plants responded to nitrogen availability by producing very green leaves due to increased chlorophyll and protein content. Furthermore, we found that PTW promotes the expression of NR and GLR1 genes, which is intensified when metals are immersed in the PTW. Furthermore, PTW and PTW with metals decreased ABI5, which is linked to growth inhibitors. The epigenetic gene expression indicates that the RONS from PTW modulates the nitrate assimilation as well as plant growth genes, which physiologically and biochemically influence the Pak Choi seedlings. However, these growth-related genes demonstrate phytohormone balancing, such as auxin production and abscisic acid catabolism, resulting in different phases of plant development. Moreover, the addition of metals to PTW neutralizes its pH and may have greater favorable impacts on plants. These findings are useful for the field of plasma agriculture to expand the use of PTW on pH-sensitive plants. The plants are sensitive to pH based on the nature of soil pH, which makes it possible for nutrients and minerals to be sustained in the soil. The most favorable pH for most plants is a neutral pH.

## Figures and Tables

**Table 1:** Physiochemical properties of DI water conditions, such as Control (DI), DI + M (no str.), DI + M (str.), PTW (str.), and PTW + M (str.), respectively. Student’s *t*-test was applied to check significance where ^a^ *p* < 0.05; ^b^ *p* < 0.005, ^c^ *p* < 0.0001.

Treated Water	pH	ORP (mV)	TDS (ppm)	Ec (µS/cm)	NOx (µM)	H_2_O_2_ (µM)
Control (DI)	6.19 ± 0.13	170.5 ± 0.707	8.8 ± 0.28	11.7 ± 1.61	0.623 ± 0.59	0.437 ± 0.02
DI + M (no str.)	7.17 ± 0.34 ^b^	176.5 ± 3.53	16.9 ± 1.27	21 ± 2.83 a	0.52 ± 0.04	0.417 ± 0.01
DI + M (str.)	8.04 ± 0.51 ^b^	194 ± 5.65	26.5 ± 1.31 ^a^	33.7 ± 1.62 ^b^	0.452 ± 0.2	0.447 ± 0.03
PTW (str.)	3.97 ± 0.065 ^c^	301 ± 1.41 ^c^	51.6 ± 1.44 ^c^	74 ± 2.12 ^c^	130.61 ± 1.39 ^c^	4.5 ± 0.26 ^a^
PTW + M (str.)	6.807 ± 0.210 ^b^	241.5 ± 0.70 ^c^	30.2 ± 1.74 ^c^	43.3 ± 2.99 ^c^	144.37 ± 1.48 ^c^	3.875 ± 0.441

## Data Availability

The data that support the findings of this study are available from the corresponding author upon reasonable request.

## Supplementary Materials

The following supporting information can be downloaded at: https://www.mdpi.com/article/10.3390/ijms24098426/s1.
