# Supplementary material for: Effect of Plasma-Treated Water with Magnesium and Zinc on Growth of Chinese Cabbage
Source: Int J Mol Sci. 2023 May 8;24(9):8426. doi: 10.3390/ijms24098426 (PMC10179069; doi:10.3390/ijms24098426)
Supplement: Supplementary file 1 [file ijms-24-08426-s001.zip › ijms-2314840-supplementary.pdf]

## Supplementary information

### Materials and methods

For the Pakchoi plants after 42 days old seedlings the shoot diameter (thickness) was measured by a micrometer screw gauge in mm scale. The measurement of shoot diameter was based on the average of ten plants per condition separately.

### Results

The shoot diameter is the point of leaf appendages. The results demonstrated that the PTW+ M (str.) diameter of the shoot is 2.439 mm which is highly significant ($p<0.001$) as compared to the control. The PTW has 1.812 mm which has a significant level of ($p<0.01$) and DI + M (str.) has 1.353 mm, with a significance of ($p<0.05$) and DI + M (no str) has 1.268 mm, which is the non-significant difference by comparing with DI which has the diameter 1.255 mm as revealed clearly in Figure S1.

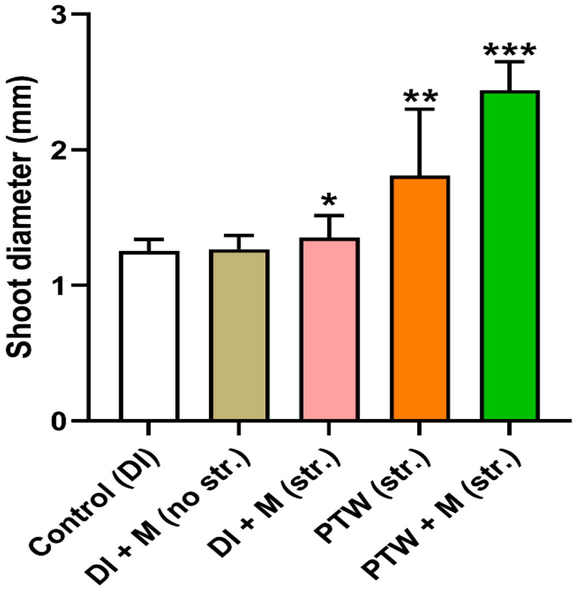


**Figure S1.** The shoot diameter of Pakchoi seedlings. The results shown in terms of error bars, and significant difference was examined by the student t-test, p-value symbolized by $^{*}p < 0.05$, $^{**}p < 0.01$, and $^{***}$ $p < 0.001$.

**Materials and methods**

For the Pakchoi plants after 42 days old seedlings the root diameter (thickness) was measured by a micrometer screw gauge in mm scale. The measurement of root diameter was based on the average of plants per condition separately. Moreover, the diameter of the roots measured from the point just below the shoot shows the maximum strength of the roots in each condition.

**Results**

The results demonstrated that the PTW+ M (str.) diameter of the root is 2.016 mm which is highly significant ($p<0.001$) as compared to the control. The PTW has 1.623 mm which has a significant level of ($p<0.01$) and DI + M (str.) has 1.312 mm, with a significance of ($p<0.05$) and DI + M (no str) has 1.31 mm, which is the non-significant difference by comparing with DI which has the diameter 1.208 mm as shown in Figure S2.

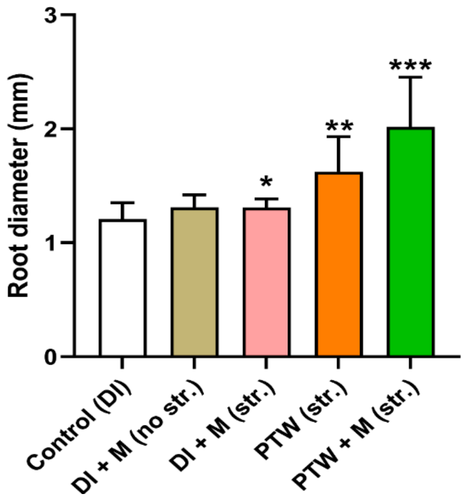


**Figure S2.** The root diameter of Pakchoi seedlings. The results shown in terms of error bars, and significant difference was examined by the student t-test, p-value symbolized by *$p < 0.05$, **$p < 0.01$, and *** $p < 0.001$.

**Materials and methods**

For the Pakchoi plants after 42 days old seedlings the number of leaves was counted. The number of leaves was counted based on physiological appearance which evaluated the physiological parameters. The number of leaves was counted separately on the average of plants per condition.

**Results**

The number of leaves displays the physiological growth of Pakchoi plants based on distinct irrigation. The results demonstrated that the PTW+ M (str.) number of the leaf is 6.6 which is highly significant (p<0.001) as compared to the control. The PTW has 6.2 which has a significant level of (p<0.01) and DI + M (str.) has 5.9, with a significance of (p<0.05) and DI + M (no str) has 5.4, which is the non-significant difference by comparing with DI which has 5 leaf number as shown in Figure S3.

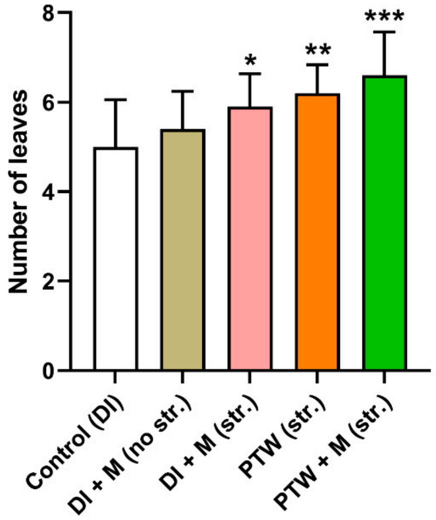


**Figure S3.** The leaf number of Pak Choi seedlings. The results shown in terms of error bars, and significant difference was examined by the student t-test, p-value symbolized by *p < 0.05, **p < 0.01, and *** p < 0.001.

**Materials and methods**

For the plant growth analysis, equally germinated seeds in DI water were planted in 50 g of vermiculite in the triplicate experiment which is ten in doublet number. Each pot has two equally germinated seeds. After this, the Pak Choi seedlings were irrigated with 10 ml of each condition freshly prepared PTW (str.), and PTW + M (str.), twice a week. At the end of six weeks (42 days old seedlings) then, the plants were gently pulled out from the vermiculite, and various physiological parameters were observed.

**Results**

The physiological status of Pak Choi plants was observed in 42 days old seedlings due to the distinct irrigation. The photographs also demonstrated that the PTW+ M (str.) shows more physiological appearance than PTW irrigation and then DI + M (str.) and DI + M (no str) have no difference as compared to the control irrigation water condition, as shown in Figure S4.

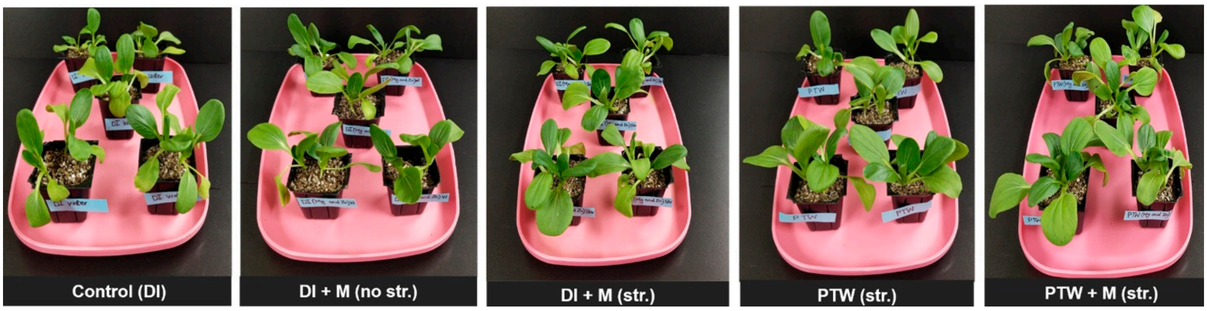


**Figure S4.** The photographs of the Pak Choi plants showed the physical growth appearance which was taken on 42 days old seedlings under the following conditions (DI), DI +M (no str.), DI +M (str.), and PTW (str.), and PTW +M (str.).

**Table S1.** Seed germination comparison within the treatment

| Comparison | Day 2 | Day 3 | Day 4 | Day 5 |
|---|---|---|---|---|
| C1 < C2 | 0.28515 | 0.39564 | 0.24009 | 0.756185399 |
| C1 < C3 | 0.00103 | 0.00057 | 0.00238 | 0.218592961 |
| C1 < C4 | 2.8E-05 | 2.7E-06 | 9.1E-05 | 0.218592961 |
| C1 < C5 | 2.8E-06 | 4.1E-07 | 1.8E-08 | 0.011607939 |
| C2 < C3 | 0.00057 | 0.00133 | 0.05372 | 0.296102008 |
| C2 < C4 | 3.4E-05 | 9E-07 | 0.00035 | 0.296102008 |
| C2 < C5 | 3.3E-06 | 5.2E-07 | 1.5E-05 | 0.008895091 |
| C3 < C4 | 0.00589 | 0.00025 | 0.00544 | 5.36754E-07 |
| C3 < C5 | 0.00025 | 6E-05 | 4.4E-05 | 2.53982E-07 |
| C4 < C5 | 0.03594 | 0.1226 | 0.04518 | 0.052653031 |

Supplementary table S1: Significance comparison within Control (DI) and treated condition C1 = Control (DI), C2 = DI + M (no str.), C3 = DI + M (str.), C4= PTW (str.), C5 = PTW + M (str.). The statistical analysis was examined by the student t-test, p – value symbolized by * $p < 0.05$, ** $p < 0.01$, and *** $p < 0.001$.

**Table S2:** List of primers used for gene expression studies.

| Gene | Sequence (5′>3′) | Sequence (5′>3′) | Length |
|---|---|---|---|
| Ethylene response factor (ERF) | CAATTCATCAGCGTTTGACG | Plus | 20 |
| | CAGCGAACTATTGTGGCTGA | Minus | 20 |
| Nitrate reductase (NR) | ATGTTCTCCATGTCCGAGGT | Plus | 20 |
| | TGATCCAAGCAGAGTCAGCA | Minus | 20 |
| Glutamate receptor 1 (GLR1) | CATTCCAACACCGAGGAAGT | Plus | 20 |
| | AACTCACGGAAGGTGAATCG | Minus | 20 |
| Abscisic acid-insensitive 5 (ABI5) | ACCACCGCATGATCAAGAAC | Plus | 20 |
| | GGTGGTTTAGTTCGGCTTCA | Minus | 20 |
| Cytochrome P450 abscisic acid 8′hydroxylase (CYP707A2) | CATCTTTGCAGCTCGAGACA | Plus | 20 |
| | CACTGGGATTTTCTCCAAGG | Minus | 20 |
| Transport inhibitor response 1 (TIR1) | TTCGTCTTACACGTGGCTTG | Plus | 20 |
| | TCGCTAACCACCATCCTCTT | Minus | 20 |
| Coupling element 1 (CHO1) | CCTCAACGACGACAACAAGA | Plus | 20 |
| | GGTCGGAAGAGAAGACAACG | Minus | 20 |
| (ARABIDOPSIS SKP1-LIKE) ASK | CTTTTGGGTCAGCCATTGTT | Plus | 20 |
| | CTTCCCGAGTTGGAGTACCA | Minus | 20 |
